# Supplementary figures and images for: RIP-seq reveals LINE-1 ORF1p association with p-body enriched mRNAs
Source: Mob DNA. 2021 Feb 9;12:5. doi: 10.1186/s13100-021-00233-3 (PMC7874467; doi:10.1186/s13100-021-00233-3)

**(A) Input RNA**

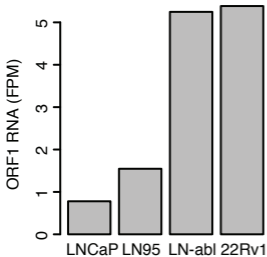

**(B) ORF1p IP**

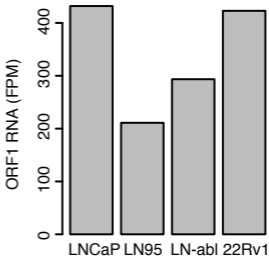

Supplement: Supplementary file 1 — Additional file 1: Figure S1. (A) LINE-1 RNA in the input for each cell LINE-1. (B) LINE-1 RNA after ORF1p-IP enrichment. [file 13100_2021_233_MOESM1_ESM.pdf]

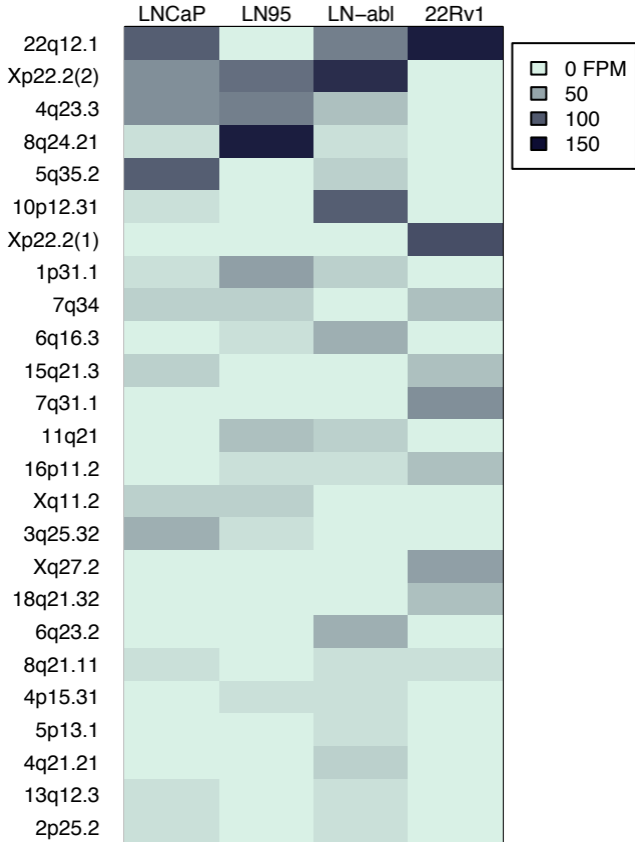

Supplement: Supplementary file 2 — Additional file 2: Figure S2. Heatmap of the specific intact loci immunoprecipitated in each cell line. Expansion of Fig. 1b to include the top 25 loci. [file 13100_2021_233_MOESM2_ESM.pdf]

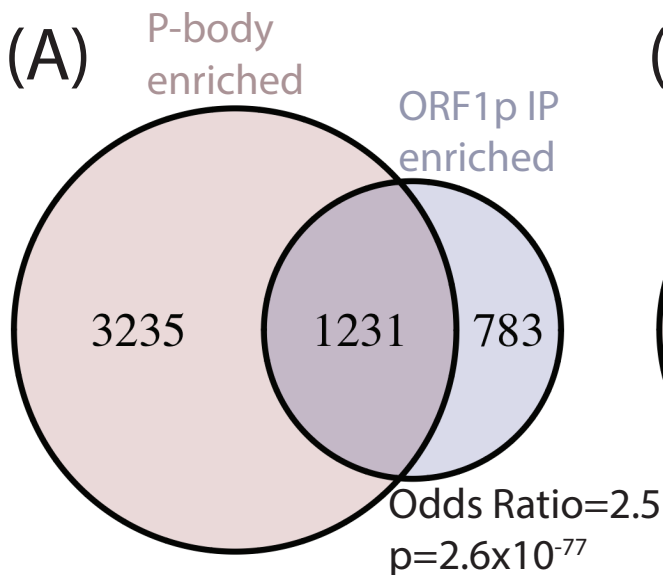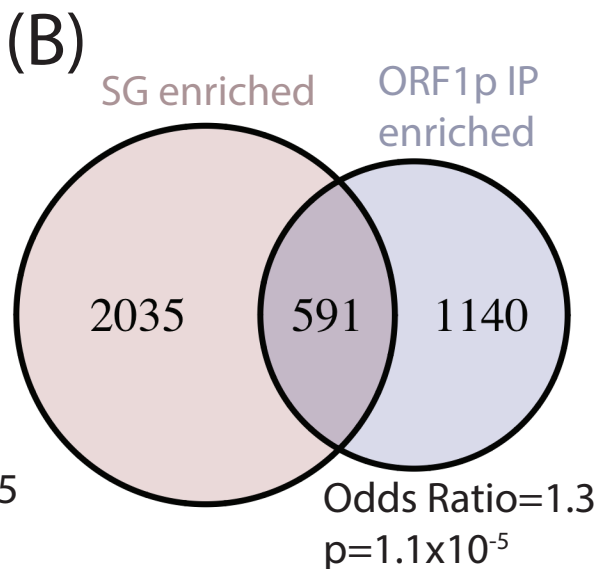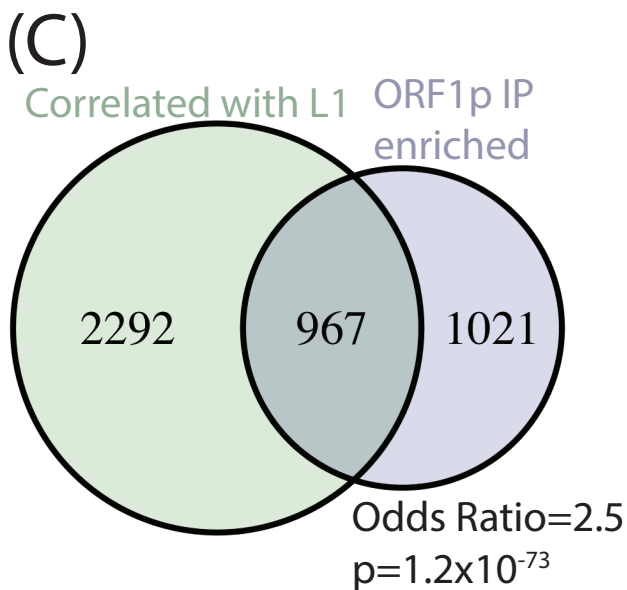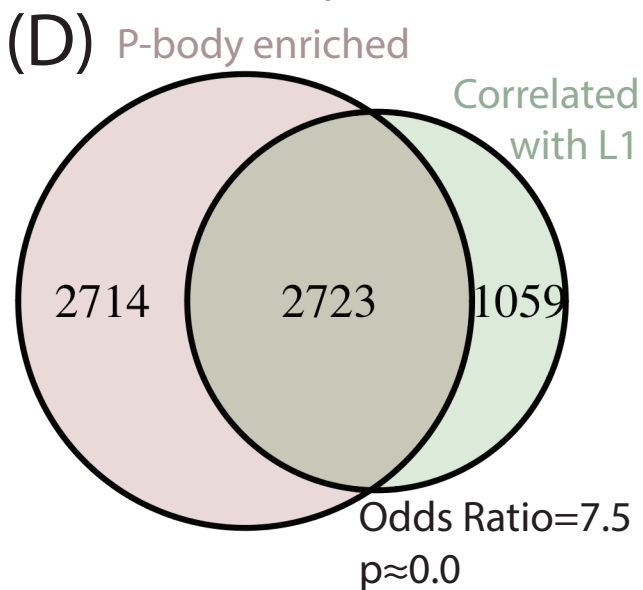

Supplement: Supplementary file 3 — Additional file 3: Figure S3. Overlap between transcripts enriched in ORF1p IP and in cytoplasmic granules. (A) Overlap between ORF1p IP enrichment and p-body enrichment [55]. A 5% FDR cutoff was used. (B) As in A, but comparing ORF1p IP to SG enrichment [54]. (C) Overlap between transcripts enriched in ORF1p IP and those whose expression is positively correlated with LINE-1 RNA in TCGA prostate cancer samples (i.e. these genes are more highly expressed in tumors that express more LINE-1). (D) Overlap between transcripts enriched in p-bodies and those correlated with LINE-1 RNA in TCGA prostate cancer. [file 13100_2021_233_MOESM3_ESM.pdf]

**A**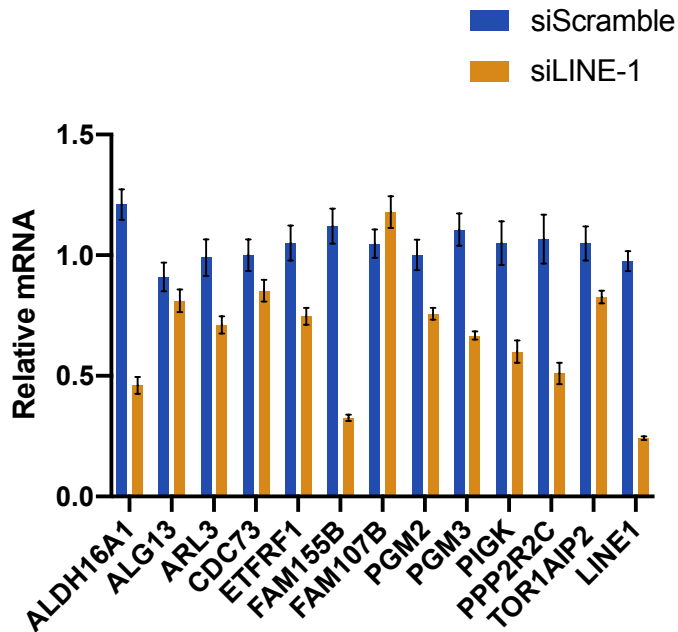**B**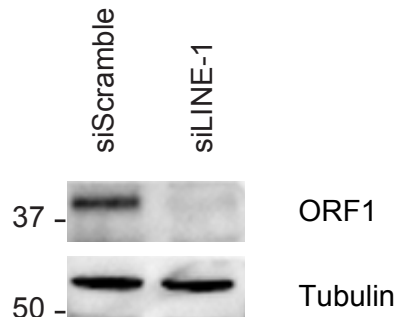

Supplement: Supplementary file 4 — Additional file 4: Figure S4. qPCR quantification of p-body and ORF1 IP RNA after siRNA knockdown of LINE-1. (A) qPCR of top ORF1 IP and p-body genes after 48 h siRNA LINE-1 knockdown. Normalized using RPL19. (B) Western blot of 48 h siRNA LINE-1 knockdown used for qPCR in (A). Demonstrates knockdown of ORF1p protein. [file 13100_2021_233_MOESM4_ESM.pdf]
